# Development of Food Chemistry, Natural Products, and Nutrition Research: Targeting New Frontiers

**DOI:** 10.3390/foods9040482

**Published:** 2020-04-12

**Authors:** Antonello Santini, Nicola Cicero

**Affiliations:** 1 Department of Pharmacy, University of Napoli Federico II, Via D. Montesano 49, 80131 Napoli, Italy; 2 Department of Biomedical and Dental Sciences and Morphofunctional Imaging, University of Messina, Polo Universitario Annunziata, 98125 Messina, Italy; ncicero@unime.it

**Keywords:** foods, food analysis, nutrition, natural products, food supplements, nanocompounds, nutraceuticals

## Abstract

The Special Issue entitled: “Development of Food Chemistry, Natural Products, and Nutrition Research” is focused on the recent development of food chemistry research, including natural products’ sources and nutrition research, with the objectives of triggering interest towards new perspectives related to foods and opening a novel horizon for research in the food area. The published papers collected in this Special Issue are studies that refer to different aspects of food, ranging from food chemistry and analytical aspects, to composition, natural products, and nutrition, all examined from different perspectives and points of view. Overall, this Special Issue gives a current picture of the main topics of interest in the research and proposes studies and analyses that may prompt and address the efforts of research in the food area to find novel foods and novel applications and stimulate an environmentally-friendly approach for the re-use of the by-products of the agro-food area. This notwithstanding, the main challenge is currently addressed to achieve a full comprehension of the mechanisms of action of food components, the nutrients, outlining their high potential impact as preventive and/or therapeutic tools, not only as a source of macro- and/or micro-nutrients, which are necessary for all the metabolic and body functions.

The complete understanding of food matrices encompasses the analytical aspects and the composition analysis, both of which are of paramount importance considering that emerging new technologies and techniques in food analysis, chemometric techniques, and methods for food authentication can allow obtaining a great amount of accurate and precise data. This potentially affects the changes in consumer preferences and expectations, as well as the analysis of food innovations and their impact on the global market [1,2].

Nonetheless, the frontier of the food chemistry has impacts with new challenges, which range from: (i) novel foods; (ii) how adequate food safety may be determined; (iii) how nutritional intakes evolve over time and are influenced by global dynamics; (iv) the novel delivery systems of the food containing health beneficial compounds, which can have a great impact on health conditions, besides their nutritional value and importance; (iv) natural sources and their waste or by-products’ recovery and re-use [3,4].

The challenge to understand the mechanism of action of food micronutrients and of the secondary metabolites involved in the chemistry of the food, especially when it is ingested, is currently triggering the interest of researchers worldwide. The complete understanding of the metabolic pathway of foodstuffs, which are complex matrices formed by many different substances, as well as the complete comprehension and assessment of the effects that food has on the body’s metabolism are still open challenges.

The analytical details and knowledge of all the minor food components and/or contaminants of different origins at a very high resolution give important information, especially on food safety and quality parameters. Nonetheless, the actual perspectives of the research in food chemistry reveal an emerging interest in a new interdisciplinary approach that involves the contribution from different disciplines both in the food and natural products areas. Natural products are and have been a primary source in many cases, not only of nutrients, but also of remedies for millennia. An example is the growing interest towards the re-use of by-products from industrial processing of food and foodstuff to recover biologically-active substances to obtain derived products originating from food matrices and that may be useful to support/supplement the diet. Nutraceuticals are an outstanding example of this emerging trend in the food chemistry area. The use or re-use of food industry by-products, as well as the recovery of biologically-active compounds are receiving growing attention in view of the great interest towards the green economy and the optimization of the available resources. In this perspective, foodstuff and agro-food industry by-products’ re-use can play a major role. A new challenging opportunity is to explore and substantiate with detailed chemical composition data and clinical data the mechanisms and modes of action of the active substances contained in food.

These aspects are relevant for maintaining well-being and preventing, by their use, the onset of diseases due to poor diet/food habits [5,6,7,8,9,10,11,12,13,14,15,16]. Safety is also a major challenge, as well as obtaining the complete or increased bioavailability of substances derived from food. From this perspective, the interest towards nanomaterials is emerging. Due to their remarkable properties, these are currently considered novel emerging tools to be used in the food area [17]. An interesting work has been reported recently regarding nanomaterials’ application to foodstuff and outlining possible beneficial effects on health [18].

Nanopharmaceuticals can be considered as an illuminating example, which have led to a great change in the pharmaceutical industry and have had a great impact also on nutraceuticals. There is increasing growth in the study of nanocompounds including nutraceuticals derived from food matrices as phytocomplexes to obtain improved delivery, bioavailability, and effects. As a consequence, many recent research works are addressed towards the use of nanotechnologies applied to food-derived nutraceuticals, building up the innovative area of new emerging products: nanonutraceuticals [19,20].

Nanotechnology could be used for the proficient delivery of bioactive substances contained in food with the aim to improve their bioavailability, thereby increasing the possible health benefits. The advantages of nanotechnology applied to nutraceuticals are efficient encapsulation, smart delivery to the target, and release from a nanoformulation. For instance, research on the encapsulation of nutraceuticals into biodegradable, environmentally-friendly nanocarriers is ongoing to increase their absorption and therapeutic potential [21].

Nanonutraceuticals are a promising tool and a new frontier for the future research in the food area, widening the horizon of foods to a new perspective, focusing on the active substances contained in food matrices and on complete understanding of their mechanisms of action in the body. These food-derived novel compounds should be assessed in order to maintain their properties at the nano level to target better bioavailability and efficacy, naturally, notwithstanding the due attention to guarantee both safety and efficacy. Follow-up studies, as well as clinical and nutritional studies to evaluate possible unwanted effects would be necessary, and there is a long way to go in targeting the above-mentioned points [22,23,24,25,26,27].

The papers that make up the Special Issue cover a wide range of topics. The application of an innovative analytical technique based on Nuclear Magnetic Resonance (NMR) experiments called Multi-Assignment Recovered Analysis (MARA)-NMR to extra-virgin olive oil allowed the quantitative assessment of the oil’s chemical composition, opening a wide range of applications [28].

The study of the Fatty Acid (FA) profile of wild *Theba pisana*, *Cornu aspersum*, and *Eobania vermiculata* land snail samples, examined by Gas Chromatography with a Flame Ionization Detector (GC-FID), put into evidence a high content of Polyunsaturated Fatty Acids (PUFAs), indicating their potential as functional food constituents [29].

The study of the native carotenoid composition in kumquat (*Fortunella margarita*) from Brazil determined for the first time by a HPLC-DAD-APCI/MS (High Performance Liquid Chromatography-Diode Array Detector-Atmospheric Pressure Chemical Ionization/Mass Spectrometry) allowed identifying and quantifying eleven carotenoids, some present in the free form and some in their esterified form [30].

The Special Issue includes studies addressing natural compounds and essential oils. In particular, nutmeg *(Myristica fragrans*) has been studied with the aim of comparing the antioxidant, antimicrobial, and anti-inflammatory activity of the hydrolats and essential oil obtained by hydrodistillation in the presence and absence of magnesium aluminometasilicate as an excipient [31].

The essential oil obtained from *Maclura tricuspidata* fruit revealed the relevant antioxidant activities of the steam-distilled essential oil and the glycosidically-bound aglycone fraction when studied with the Gas Chromatography–Mass Spectrometry (GC–MS) technique [32].

Functional food ingredients were exploited in the study on *Uraria crinita* by screening its metabolites using immunomodulatory fractions from the root methanolic extract in combination with bioactivity-guided fractionation and NMR-based identification [33].

Other manuscripts published in the present Special Issue evaluated the capacity of Elderberry fruit (EDB) extract to decrease the elevated production of reactive oxygen species in hypertrophied 3T3-L1 adipocytes, evidencing a crucial role in the development of obesity and accompanying metabolic dysfunctions [34].

A study on the sea tangle (*Laminaria japonica* Aresch), a brown alga, used as a functional food ingredient in the Asia-Pacific region, allowed assessing how fermented sea tangle extract was effective on the receptor activator of the nuclear factor-κB (NF-κB) ligand using RAW 264.7 mouse macrophage cells [35].

In addition, another interesting study contained in the Special Issue evaluated the antioxidant and anti-adipogenic activities of another vegetal matrix, namely a mixture of *Nelumbo nucifera* L., *Morus alba* L., and *Raphanus sativus*, with a complete updated in vitro and in vivo study [36].

The anti-inflammatory potential effect of plant sterols from enriched milk-based fruit beverages (with or without galactooligosaccharides in an experimental mouse model of chronic ulcerative colitis) was proposed, evidencing a great beneficial effect in mice against colitis [37].

Along the same lines, another interesting paper addressed foods in traditional medicine with antioxidant potential, in particular the assessment of the antioxidant effect of leaf extracts of *Solanum nigrum* L. [38].

The topics of the Special Issue expand the horizon of food research, also examining other applications of vegetal matrices. An example is the paper dedicated to the study of Llayta, a biomass of the colonies of *Nostoc cyanobacterium* grown in the wetlands of the Andean highlands, harvested, sun-dried, and used as an ingredient for human consumption, which revealed great potential as a functional food ingredient due to its relevant content of essential amino acids and polyunsaturated fatty acids [39].

The collection of papers is completed with one study addressing also industrial food applications, especially for industries interested in replacing artificial dyes with natural pigments; cyanobacterial phycobiliproteins as water-soluble colored proteins to be used as natural eco-sustainable pigments were shown to have great potential in this area of interest [40].
